# Can ResilienceNHope, an evidence-based text and email messaging innovative suite of programs help to close the psychological treatment and mental health literacy gaps in college students?

**DOI:** 10.3389/fpubh.2022.890131

**Published:** 2022-10-28

**Authors:** Belinda Agyapong, Reham Shalaby, Yifeng Wei, Vincent I. O. Agyapong

**Affiliations:** ^1^Department of Psychiatry, University of Alberta, Edmonton, AB, Canada; ^2^Global Psychological eHealth Foundation, Edmonton, AB, Canada; ^3^Department of Psychiatry, Faculty of Medicine, Dalhousie University, Halifax, NS, Canada

**Keywords:** resilience, hope, mental health, anxiety, depression, stress, text messaging

## Abstract

There is a high prevalence of stress, anxiety, depression, and substance use disorders in college students globally. Financial stressors, course workload, peer pressure, and other personal, family, and societal stressors contribute to the high incidence of mental disorders among college students. Despite the high prevalence of mental disorders in college students, barriers such as lack of mental health literacy, stigma of mental health, inadequate numbers of mental health counselors and clinical psychologists supporting students in colleges in both low- and high-income countries, and financial and geographical barriers often hinder college students from accessing the needed mental supports. There is increasing evidence on the effectiveness and feasibility of mobile technology in health promotion and closing psychological treatment gaps. College students are well adapted to the use of mobile technology, particularly text and email messaging daily, which presents a unique opportunity for an innovative way to offer support for their mental health. In this article, we provide a perspective on the ResilienceNHope program, an evidence-based text and email messaging innovation, to close the psychological treatment gap and improve the mental health literacy of college students.

## Introduction

Higher education students experience many challenges leading to increased vulnerability and hence a high incidence of mental health issues. The education and learning of 67.6% of students are impacted worldwide due to the COVID-19 pandemic (1). Globally, many studies have confirmed the high prevalence of mental health issues among college or university students. A cross-sectional study among university students found that 27.5% had moderate and 9.7% had severe or extremely severe depression; 34% had moderate and 29% had severe or extremely severe anxiety; and 18.6% had moderate and 5.1% had severe or extremely severe stress (2). Another study showed that the prevalence of depressive symptoms was 32.6% (3), while anxiety and depressive symptoms were associated with lower grades (4). In another cross-sectional study of university students, more than half of the participants were affected by depression (51.3%), anxiety (66.9%), and stress (53%) (5). Results of another study reported that 34.6% had depressive symptomatology, 37.2% showed anxiety symptoms, and 47.1% had stress symptoms (6).

The ongoing pandemic and online learning has exacerbated the mental health burden of the university student. Approximately one-third of college students experienced varying degrees of depression, anxiety, and stress symptoms with online learning during the COVID-19 pandemic (7). One study reported that isolation measures to curb the COVID-19 pandemic including online learning, mostly home-based learning, had a huge health impact with the potential to affect long-term diet and physical activity in both sexes with approximately 30% fewer students achieving adequate levels of activity, compared with the previous 2 years (8). The lack of campus time and face-to-face learning contact can also result in learners feeling isolated (9) and has significantly disrupted the lives of students (1). One study reported that students experienced increased stress and anxiety as well as difficulties concentrating during online learning (10). Acute stress, anxiety, and depressive symptoms have been prevalent among students during the COVID-19 epidemic (11). One study found the prevalence of depression, anxiety, and stress were 31.9, 32.9, and 14.6%, respectively (7). Another study reported the prevalence of moderately severe to severe depression and anxiety were 34.3% and 20.1%, respectively (12).

The stressors experienced by university students in Canada are not different from those experienced by students in other countries. A cross-sectional survey in Canada reported 39.5% symptoms of moderate to severe depression, 23.8% of moderate-severe anxiety, and 80.3% of moderate-severe levels of perceived stress (13). In addition, over the academic year, 14% of students reported suicidal thoughts and 1.6% suicide attempts (4). Moderate to severe levels of anxiety and depressive symptoms persist among university students, especially in the first year and the use of drugs seems to increase these risks (14). A longitudinal study of students on entry showed clinically significant depressive symptoms (28%) and anxiety symptoms (33%) which increased to 36 and 39%, respectively, by the end of the first year (4).

The high prevalence of toxic stress, anxiety, depression, and substance use disorders can be attributed but not limited to factors such as financial stressors, course workload, peer pressure, and other personal, family, and societal stressors (15). A survey of university students reported lack of money, time management, coursework assessment items, lack of sleep, and course marks as having a major impact on their mental health (16). The prevalence of anxiety seems to be much higher than either depression or stress (2), which can be explained by the uncertainties of life coupled with course workload. Depressive symptoms are significantly related to grade point average and examinations or tests (3, 13). Anxiety about examination generally results in depression, excessive worry, and stress about the evaluation of tests and results (17). Anxiety can also be a result of socioeconomic factors or family factors (13). Financial stressors also contribute immensely to the mental health burden and a cross-sectional survey suggest that there are significantly higher stress scores among students whose family had low incomes (2).

The risk of alcohol or drug use among university students is also due to the substantial changes occurring in their lives, including social factors, peer, teachers, or parent's pressure (18). A cross-sectional study of university students in Nigeria showed that nearly 4.2% were dependent on alcohol, while 14.1% had “low-risk use” for other psychoactive substances (19). In another study conducted in Switzerland, a slightly higher percentage (20%) of university students reported increased alcohol consumption and 26% engaged in binge drinking (20).

In contrast to the wide availability of psychotropic medications for the treatment of mental disorders (21), psychological treatment services are usually limited or completely unavailable. Barriers to access alongside inadequate resources have contributed to the limitations in providing or making these services available to patients with mental illness, thus creating a therapeutic gap.

Mounting evidence report the global lack of mental health professionals, including therapists, particularly in remote areas and rural communities in addition to the costly and resource-intensive nature of the conventional supportive services (21–25). Besides, there are other personal beliefs and culture-based factors such as the stigma or discrimination barriers emerging from the social or professional circles surrounding the patients that may limit the accessibility to these services and may further contribute to enlarging the therapeutic gap (21–25).

A large body of literature, therefore, has suggested that remotely delivered services could be comparable to face-to-face services and effective in addressing mental health conditions (23, 26). These services have been recently adopted in the healthcare system allowing for the use of technology and the internet to either communicate with a healthcare provider or instead to deliver the health services and information *via* mobile devices such as smartphones using SMS, MSS, mobile, or web applications (27–30). The provision of easily accessible interventions that require the patients to use an app or internet program with no human interaction was compared against therapist involvement in an intervention (27, 31). Phone chat or texting was the preferred mode of communication among adolescents with their physicians, compared to face-to-face (32). Adolescents usually report more convenience with reduced anxiety and effective communication. Therefore, the growing spread of such wireless services in the healthcare system has become more reasonable among young adults.

## ResilienceNHope

ResilienceNHope (33) is an evidence-informed e-mental health application which delivers one-way (non-interactive) psychological interventions which incorporate cognitive behavioral therapy based on daily supportive messages (mobile text or email), weekly mental health literacy information, online mental health self-assessments, and other mental health resources to help address part of the mental health literacy and the mental health treatment gap for individuals and communities globally. Subscribers are informed of the non-interactive nature of the supportive messaging program through the welcome and introductory messages they receive on subscribing to the program. They are also offered the phone number of the mental health crisis service for their province or region to call if they are in crisis, such as if they experience suicidal ideation. In addition to the English programs, ResilienceNHope mobile text-based program include Arabic, French, Punjabi, Mandarin, Ukrainian, and Russian languages (34) and can easily be adapted to other languages. Both the email and text message programs aim to support regional and national health authorities and institutions seeking to implement evidence-based, cost-effective, and easily scalable population-level e-mental health programs. The current suite of programs includes Text4Mood, Text4Support, Text4Hope, Text4HopeAddiction Support, Text4Hope Cancer Care, Text4PTSI, MoreGoodDays, Positive Mental Health, ResilienceNHope4Ukraine, and Wellness4Teachers as described in Table 1.

**Table 1:** Description of the ResilienceNHope suite of programs.

**Program**	**Organization/date launched**	**Province/country**	**Program description**
Text4Mood (35)	Alberta Health Services, 2016	Alberta	Address stigma, long waitlists, and geographical barriers to access to counseling services for anxiety and depression. The program was recognized by the Mental Health Innovations Networks which is headquartered at the Department of Mental Health and Substance Abuse of the World Health Organization. Over 25, 000 As at the end of February 2022,
Text4Support (36)	Alberta Health Services, January 2018 Scheduled for launch by Nova Scotia Health Authority and Dalhousie University as part of a clinical trial in July 2022	Alberta and Nova Scotia	Provides mental health diagnosis specific supportive text messages to addiction and mental health patients who are receiving formal face-to face mental health services. Programs under Text4Support include Depression Program, Anxiety Program, Bipolar Program, Psychosis Program, Addiction Program, Trauma Program, Personality Disorders Program, Eating Disorders Program, and an Obsessive-Compulsive Disorders Program.
Text4Hope (37)	Alberta Health Services, March 2021	Alberta and British Columbia, Canada	Provide support for individuals experiencing stress, anxiety, and depression because of the COVID-19 pandemic. Available in English, French, Punjabi, Mandarin and Arabic, Text4Hope has had over 55,000 subscribers as at the end of February 2022
Text4Hope Addiction Support (38).	Alberta Health Services, March 2021	Alberta, Canada	To provide addiction related supportive text messages for individuals dealing with addiction problems during the COVID pandemic
Text4Hope Cancer Care (39).	Alberta Health Services, March 2021	Alberta, Canada	to provide psychological support for patients undergoing cancer treatments during the COVID pandemic
Text4PTSI (40).	University of Alberta, June 2021	Alberta, Canada	Provide psychological support for first responders experiencing post-traumatic stress injury symptoms
MoreGoodDays (41)	Alberta Kickstand, June 2020	Alberta, Canada	Provide psychological support for the youth and young adults. Messages are crafted by young people and reviewed by a clinical team.
Positive Mental Health Program (33)	Global Psychological eHealth Foundation. Scheduled for launch as a global program in December 2022	Worldwide	School/college and workplace based mental health literacy and supportive email and voice messaging program
Welness4Teachers (42)	Global Psychological eHealth Foundation. Scheduled to be launched in September 2022	Alberta and Nova Scotia Canada	Provide psychological support for teachers experiencing symptoms of burnout, stress, anxiety and depression
ResilienceNHope4Ukraine (43)	Global Psychological eHealth Foundation, March 2022	Canada	Provide psychological support for Canadian experiencing symptoms of stress, anxiety and depression and PTSD because of the war in Ukraine. The program is available in English, French, Ukrainian and Russian languages.

## Scientific evidence in support of the efficacy and effectiveness of ResilienceNHope messaging programs

ResilienceNHope text-based messaging programs have provided evidence as cost-effective and easily scalable programs designed to provide psychological support for individuals with various mental health conditions, including alcohol abuse, Major Depressive Disorder (MDD), Generalized Anxiety Disorder (GAD), perceived stress, Post Traumatic Stress Disorder (PTSD), sleep disorder symptoms, suicidal ideations and thoughts of self-harm, and comorbid conditions. Besides the reported effectiveness, the service has achieved expansive reachability along with the reported satisfaction among the subscribers. The text-based programs have further facilitated surveillance of mental health conditions among subscribers who are mostly mental health and addiction patients and the general public who provide serial online surveys which include questions accessing the presence of mental health symptoms.

In the following section, we will highlight the utility of the ResilienceNHope text-based programs, evidence of their effectiveness, and the potential applicability of the programs to help close the mental health treatment gap among college students.

### Drug/alcohol abuse

- A randomized controlled trial (RCT) (44) was conducted involving 59 patients with alcohol use disorders (AUD) who completed a residential addiction treatment program in Grande Prairie, Alberta, Canada. Patients in the intervention group (*n* = 29) received daily automated unidirectional, non-interactive supportive text messages crafted based on addiction counseling principles and sent through an online program to the patients' cell phones for 3 months following discharge. Patients in the control group (*n* = 30) received a text message thanking them for participating in the study. A trend was observed in the intervention group to have more than double the mean number of days to their first drink after discharge from the residential treatment program, compared to the control group (60 vs. 26 days respectively, mean difference: 34.97; 95% CI: −5.87–75.81). Small to moderate effects were found for Cumulative Abstinence Duration (CAD) and units of alcohol per drinking day. Small to negligible effects were found for health utilization. On subgroup analyses, the participants who received text messages, among those who did not attend follow-up outpatient counseling, showed a longer CAD. Additionally, patients with alcohol use disorder who received text messages had a trend toward recording larger cumulative alcohol abstinence in days.
- In another study (45), the authors examined the PTSD symptoms and its mental health associates in the residents of a Canadian community of Fort McMurray, 6 months after enduring a wildfire that forced over 90,000 residents to evacuate after the fire consumed about 2,400 homes and over 200,000 ha of forest. The authors found that the respondents who presented with likely PTSD after the wildfire were significantly more likely to self-report increased drug abuse (8.1 vs. 2.1, *p* = 0.02), but not increased alcohol use (16.4 vs. 11.4%, *p* = 0.3), compared with respondents who did not have likely PTSD.
- In an ongoing research work (38), the authors have implemented a program of daily supportive text messaging (Text4Hope-Addiction Support) to reduce drug or alcohol cravings as well as anxiety and depression, typically associated with alcohol and substance use disorders. The authors evaluated the prevalence of cravings, anxiety, and depressive symptoms; demographic correlates of the same; and the outcomes of the Text4Hope-Addiction Support intervention in mitigating cravings, anxiety, and depressive symptoms during the COVID-19 pandemic. Preliminary unpublished data reveal a significant reduction in craving intensity, craving frequency, and length of time craving drugs, after receiving a 3-month daily supportive text message (Text4Hope-Addiction support), as reported on the Brief Substance Craving Scale (*n* = 67).

## Major depressive disorder

- In an RCT involving 73 patients diagnosed with MDD, patients in the intervention group (*n* = 35) received twice-daily supportive text messages for 3 months as part of their outpatient treatment, while the control group (*n* = 38) received a single thank-you message every fortnight (46). Patients in the intervention group (20.8; SD = 11.7) had a significant greater reduction (25%) in their depressive symptom on BDI scores compared to patients in the control group (24.9; SD = 11.5), F (1, 60) = 4.83, *p* = 0.03, η*p*2 = 0.07), with a medium effect size Cohen's *d* = 0.67) (47). Furthermore, after adjusting for baseline scores, a significant difference remained in the 3-month mean self-rated VAS scores (EQ-5D-5 L scale) between the intervention and control groups, 65.7 (SD = 15.3) vs. 57.4 (SD = 22.9), F (1, 60) = 4.16, *p* = 0.05, η*p*2 = 0.065. The mean difference in change means self-rated VAS scores were also statistically significant with an effect size (Cohen's *d*) of 0.51.
- Early in the COVID-19 pandemic, Text4Hope, a service that used supportive SMS text messaging as an evidence-based, with prior research supporting good outcomes and high user satisfaction to reduce distress related to the COVID-19 crisis, initially among Canadians (37).
- After 3 months, there was a significant reduction (10.3%) in likely depressive symptoms on the Patient Health Questionnaire-9 scale score from baseline (9.32; SD = 6.23) to 3 months (8.36; SD = 6.62) (*t* (301) = 3.16, *p* = 0.002) (48).
- Similarly, subscribers of Text4Hope who had been enrolled for 6 weeks (Intervention Group) had a significantly lower prevalence of depression compared to new subscribers during the same time period (Control Group) (36.8 vs. 52.1%, respectively) (49). After controlling for demographic variables, subscribers in the intervention group remained less likely to self-report symptoms of MDD (OR = 0.50; 95% CI = 0.47–0.73), compared to the subscribers in the control group.

### Comorbid MDD and AUD

- In an RCT, patients with AUD and comorbid depression (*n* = 95) were recruited after completing a 30-day rehabilitation program. The intervention group (*n* = 47) received twice-daily supportive text messages over 6 months while control participants (*n* = 48) had treatment as usual for 6 months, with an added 6-month post-treatment follow-up for both groups. At 3 months, depression (*P* = 0.02) and perceived stress scores (*P* < 0.01) were significantly reduced in the intervention group relative to control participants with small to medium effect. A significantly greater reduction in units per drinking day (U = 494, *P* = 0.03, *r* = −0.3) from baseline to 6-month treatment point was reported in the intervention group, compared to the control group with a medium effect size (*P* = 0.03). There were no differences in drinking or mood measures at 6-month post-treatment follow-up (50). This may highlight the value of the continuity and sustainability of texting-based programs to ensure improved mental health.
- Another RCT aimed to explore the effects of supportive text messages on mood and abstinence outcomes for patients with depression and co-morbid AUD. The participants (*n* = 54) with a DSM-IV diagnosis of unipolar depression and AUD who completed an in-patient dual diagnosis treatment program were randomized to receive twice-daily supportive text messages (*n* = 26) or a fortnightly thank you text message (*n* = 28) for 3 months. At 3 months (51), there was a statistically significant difference in depression symptoms as measured on BDI-II scale scores between the intervention and control groups; after adjusting for the baseline scores, with a mean difference of – 7.9 (95% CI: −13.06 to −2.76, Cohen's *d* = 0.85). Additionally, there was a trend for a greater CAD in the text message group than the control group: 88.3 (SD = 6.2) vs. 79.3 (SD = 24.1), *t* = 1.78, df = 48, *p* = 0.08.

At 6 months, which was 3 months after the cessation of the texts, the results of the same study showed unlike at 3 months, there was no statistically significant difference in BDI-II scores or CAD between the text message group and the control group. However, patients in the intervention group had significantly higher days to first drink compared to those in the control group: 119.9 (47.7) vs. 62.4 (44.9), *t* = 2.99, df = 22, *p* = 0.01 (52).

### Generalized anxiety disorder (GAD)

- The positive impact of the daily supportive texting service, Text4Hope, on anxiety symptoms, was reported at the mid-point and end of the program as follows: there was a significant reduction (−18.7%, *p* < .001) in the Generalized Anxiety Disorder-7 (GAD-7) scale's measured mean anxiety symptom score from baseline (mean = 9.62, SD = 5.6) to 6 weeks (mean = 7.82, SD = 5.2) after receiving daily supportive messages, with a small effect size (Cohen *d*: 0.4) (53). Furthermore, the prevalence of likely generalized anxiety disorder (GAD) among subscribers was significantly reduced at 6 weeks compared to the baseline figures (45.8 vs. 32.3%, respectively).

Similar results were obtained at 3 months with a 22.7% reduction in the GAD-7 score from baseline (mean = 9.07, SD = 6.02) to 3 months (mean = 7.01, SD = 5.84) (48).

- The effectiveness of Text4Hope in combating mental health symptoms was evaluated in a comparative study, by comparing psychiatric parameters between two subscriber groups. The first group was the Text4Hope subscribers who received daily texts for 6 weeks (intervention group), while the second group was the new Text4Hope subscribers who were yet to receive messages (control group). The results revealed that the intervention group had a significantly lower prevalence of anxiety compared to the Control Group (31.4 vs. 46.5%, respectively) (49). Furthermore, the subscribers belonging to the intervention group remained less likely to self-report symptoms of likely GAD (OR = 0.55; 95% CI = 0.44–0.68), after controlling for demographic variables.

### Stress

Similar to the examined GAD symptoms, stress symptoms were examined through the Text4Hope initiative, as follows:

- There was a significant reduction in stress symptoms as measured by the Perceived Stress Scale (PSS-10) (−18.7%, *p* < 0.001) from baseline (mean = 20.35, SD = 6.7) to 6 weeks (mean = 19.51, SD = 7.0) (53), and when compared to 3 months, there was a 5.7% reduction in the PSS-10 score from baseline (mean = 20.21, SD = 7.23) to 3 months (mean = 19.07, SD = 7.74) (48). Likewise, stress prevalence among subscribers was significantly reduced at 6 weeks (5.4%) and 3 months (6.9%), compared to baseline data.
- In the aforementioned comparative study, the intervention group had a significantly lower prevalence of moderate or high stress compared to the Control Group (78.8 vs. 88.0, respectively), and the subscribers belonging to the intervention group remained less likely to self-report symptoms of stress (OR = 0.56; 95% CI = 0.41–0.75), after controlling for demographic variables (49).

### Suicidal thoughts

In the same comparative study, which was run to evaluate the effectiveness of Text4Hope, 6 months after launching the service, the subscribers in the intervention group showed a significantly lower prevalence of suicidal thoughts and self-harm ideas, compared to the control group (subscribers who were yet to receive supportive text messages) (16.9 vs. 26.6%, respectively) (49). Furthermore, intervention group subscribers remained less likely to self-report symptoms of suicidal thoughts and self-harm (OR = 0.59; 95% CI = 0.45–0.77), after controlling for demographic variables (49).

### Sleep symptoms

In the same comparative study, the intervention group had a lower prevalence of disturbed sleep compared to the control group (76·9 vs. 85.1%, respectively), although this was not statistically significant after Bonferroni correction (*p* = 0.02, which is larger than the adjusted *p* = 0.01) (49).

## Other evidence in supporting text messaging or email applications

Text messages are a current platform that incorporates technology into the healthcare system, spanning a wide range of health conditions and playing different roles. Text messages are used as a reminder of medical appointments (54) or to encourage adherence to prescribed medications (55). Supportive texting programs, such as Text2quit and Quit4baby, have been provided to people in the field of addiction and smoking, particularly during their vulnerability, achieving considerable success. Text4baby was provided to pregnant women and new mothers aiming to improve their health beliefs and attitudes (56, 57) and Text2quit was provided to adults and pregnant women to quit smoking (58, 59). Favorable response to these programs have been reported praising the content and the skills taught that helped them with positive ideas on quitting, to the extent that they may recommend it to a friend.

In the field of addiction and drug use, several studies examined the effect of text messages with reported efficacy and feasibility for such programs in combating drug and alcohol-related problems in young people. For example, Mason, Ola et al. (60), in their meta-analysis, aimed to examine the effectiveness of text message interventions for tobacco and alcohol cessation within adolescent and young adult populations. A total of 14 RCT studies were examined, and the authors reported an overall effect size of 0.25, concluding while the effect sizes varied among the studies, approximately one in three people in the treatment groups reduced their tobacco or alcohol use, compared to people in control groups. This indicated that text interventions have a positive effect on reducing substance use behaviors among the young population, and the prevention of SUD and smoking could be enhanced *via* texting services.

In another systematic review, Hutton, Prichard et al. (61) examined 15 articles regarding the effect of mobile-based health interventions (mHealth), such as social networking sites, SMS, and mobile phone applications on reducing harmful alcohol-related behaviors. Their target population was young people aged between 12 and 26 years, without known alcohol addiction or alcohol dependency. The authors reported that the service was effective in reducing alcohol consumption in 50% of their studies, with above 70% in text messages-based studies. Furthermore, the authors reported that young people liked personalized messaging that helped in an effective way to convey supportive services; and concluded that the use of mHealth, particularly text messaging, was found to be an affordable, acceptable, and effective way to deliver messages to reduce alcohol consumption in young people. With respect to the text-based and online services in the field of psychosis and schizophrenia, several studies reported positive results related to the efficacy, better engagement, and feasibility of these interventions. For example, in a recent systematic review run by *D*'Arcey, Collaton et al. (62), the authors concluded that text messages were generally safe, feasible, easy to use, and wellperceived as reported by more than half of the study participants. Additionally, the authors reported that text messages can leverage patients' engagement in terms of improving clinic attendance, better adherence to medications, and therapeutic alliance.

In another systematic review examining both the online and mobile-based interventions, Alvarez-Jimenez, Alcazar-Corcoles et al. (29) indicated that both interventions may show promise in improving positive psychotic symptoms among patients with schizophrenia-spectrum disorders. The authors reported that such interventions, particularly mobile-based, may help to monitor early relapse signs that may reduce hospital admissions. Additionally, they noticed that tailored text-based interventions were found to be associated with improved symptomatology in terms of reduced hallucination severity and improved sociability but not functional outcomes.

Similarly, several studies examined texting services in addressing depression and mental health conditions or symptoms either alone or in association with anxiety and suicidal symptoms.

In a systematic review by Cox, Allida et al. (24), the authors aimed to examine text messaging interventions in people with depressive symptoms. The review included seven trials in their review with 1,918 participants. The authors concluded that there is a significant effect of text messages in reducing depressive symptom scores, however, due to the substantial heterogeneity (high inconsistency in results), the effect was described as borderline. Considering depression as a primary outcome in this review, the sensitivity analysis reported a statistically significant reduction in depressive symptoms with a low heterogeneity in the intervention group compared to the control group at the end of treatment (SMD, −0.30; 95% CI, −0.53 to −0.08; *I*2 = 23%). Similar results were obtained while considering trials using standard depression rating scales. The review also concluded that the effectiveness of text messages in reducing depressive symptoms was achieved when two or more messages were sent per week, while a lower number may not produce a significant effect.

In another scoping review, Dwyer, de Almeida Neto et al. (63) reported on text messages in adjunction with other e-mental health as counseling services. The authors concluded that there is converging evidence that text-based counseling services and interventions are effective in treating a variety of mental health conditions, such as depression, suicidal ideation, or anxiety, particularly when patients with depression are comfortable with online communication. With respect to text-based counseling, it was found to be effective in treating depression and psychological distress, and the communications analyzed using computational linguistic techniques can be applied to identify individuals at risk of serious mental health or suicide (63).

To examine the use of text messaging-based interventions and identify technological and clinical design features of these interventions in the young population, MacDougall, Jerrott et al. (32) in their scoping review examined the literature for text-based services in mental health among children and adolescents. The authors included 31 studies providing data related to the nature, frequency, and targeted assessments of text-based services in the field of mental health. The authors observed that patients' engagement was the main outcome measure in the majority of the studies. Regarding the frequency of the interventions, it seems that a smaller number and less frequency of text messages were provided to the younger population compared to the adults, where the authors reported that text messages were usually delivered in less than 12 weeks with a range between one to three messages per week. With respect to the clinical condition, the two main targeted mental health conditions were substance use or problem drinking (35% of the studies) and depression in adolescents (32% of the studies). Most studies reported the use of bidirectional messaging system (65% of the studies), with limited data on the cost or policy implications.

## Feasibility for implementing ResilienceNHope messaging programs among college students

ResilienceNHope service has the potential to expand along the young population, including college students. The evidence based on the outcomes of the service has flagged out the high risk in the young population of experiencing mental health symptoms, such as, anxiety, Post-traumatic stress disorder (PTSD), and passive death wish and thoughts of self-harm, particularly during the COVID-19 pandemic (64–66). Izu et al. (67) compared the prevalence and the severity of different mental health conditions among Text4Hope subscribers, based on their age groups. The authors reported that 11% of the total survey respondents (*n* = 8,267) were identified as 25-year-olds or less. The mean scores on the Perceived Stress Scale 10 (68), the Generalized Anxiety Disorder 7-item (69), and the Patient Health Questionnaire-9 scale (70) were highest among this young population and lowest among those aged >60 years (25.4 vs. 16.65, 12.23 vs. 6.35, 13.05 vs. 6.65, respectively). The authors proposed some explanations, such as the younger adults may be exposed to more information about the pandemic *via* social media or their loss of social connections with friends which may render them more vulnerable to mental distress. Such alarms lead to the development of MoreGoodDays program, one of the ResilienceNHope suite of a free daily text messaging service provided by Kickstand for the youth in Alberta (41). The service provides adolescents and young adults with a 1-year daily supportive text message. Although anyone can register for MoreGoodDays, the service is primarily aimed to support the young populations and promote their mental health and wellbeing. According to the latest reports, more than one half of MoreGoodDays subscribers are college students (138/263, 52.5%). The preliminary feedback is promising, the end users among adolescents and young adults usually report their acceptance and satisfaction and further seek service extension after 1 year. This led to the provision of a second version of the service that has enabled those who are interested to re-register to the service more than one time.

The feasibility, accessibility, and reachability domains of ResilienceNHope programs was achieved among young adults. This was clearly reflected in the considerable representation of young people in the Text4Hope service. Given that the service was provided to everyone in Alberta, regardless of their age, at 1 year, there were 1,199 subscribers who identified as 25 years or less (11%), and 600, 5.7%, were students. Furthermore, the unpublished data for 1-year Text4Hope service showed that the subscribers who were ≤ 25 years achieved a better improvement (higher reduction) in the mean scores of likely stresses (−2.11 vs. −1.30) and likely GAD (−2.40 Vs. −2.11) as compared to those above 25 years, respectively, albeit the results were not statistically significant. Similarly, just less than a quarter of Text4Support, self-subscribers were young adults (18-24y) (36). Taken from these text-based services seem to be acceptable, accessible, and effective in young populations.

## Likely acceptability of ResilienceNHope messaging programs among college students: Subjective mental wellbeing and user satisfaction

Besides the reported clinical effectiveness of ResilienceNHope programs, the service has achieved an expansive acceptability and satisfaction among subscribers. To examine the perception and feedback of patients with AUD and comorbid depression about the usefulness of supportive text messages, a randomized trial was designed with participants who have a DSM-IV diagnosis of AUD and depression (*n* = 26), and who completed an in-patient dual diagnosis treatment program were provided with twice-daily supportive text messages delivered to their mobile phones for 3 months (71). At 3 months, 18 (75%) patients reported that the text messages always or often reminded them to remain abstinent from alcohol. Twenty (83%) patients reported that the intervention had played a useful role in helping to improve their mental health, in particular, in serving as a motivation for recovery and in preventing relapse.

Subscribers of Text4Mood program (text-based program that delivers daily supportive text messages to subscribers was launched in 2016) (*n* = 894) reported that daily supportive messages made them feel more hopeful about managing issues in their lives (82 %, *n* = 588), in charge of managing depression and anxiety (77 %, *n* = 552), coping with stress (77 %, *n* = 552), and feel connected to a support system (75 %, *n* = 542). Most subscribers also reported that Text4Mood improved their overall mental wellbeing (83 %, *n* = 598), made them feel like they could bounce back if they made a mistake (77 %, *n* = 554), and 52% (*n* = 461) reported that the daily messages helped them elevate their mood. Furthermore, most subscribers felt the daily messages were positive (98%), supportive (95%), on topic (88.5%), and to the point (87%) (72). Similar results were obtained from Text4Hope service (the mental health service provided by health authorities in Alberta, Canada, to support the mental wellbeing and distress associated with the CVID-19 pandemic) (37). More than 70% of the subscribers agreed that Text4Hope helped them cope with stress (1334/1731, 77.1%) and anxiety (1309/1728, 75.8%), feel connected to a support system (1400/1729, 81%), manage COVID-19-related issues (1279/1728, 74%), and improve mental wellbeing (1308/1731, 75.6%). Similarly, subscribers agreed that messages were positive, affirmative, and succinct. Messages were always or often read by 97.9% (1681/1716) of respondents, and more than one in five (401/1716, 23.4%) reported that they always or often returned to messages. Most subscribers (1471/1666, 88.3%) read the messages and either reflected upon them or took a positive action (73). Furthermore, the subscribers were asked about their acceptance of technology-based services in supporting their health. Most subscribers welcomed almost all technology-based services as part of their health care (mental or physical), during crisis, or emergency situations (73).

## Conclusion

College students are confronted by multiple stressors and are therefore vulnerable to experiencing psychological disorders such as anxiety, depression, and substance use disorders. This notwithstanding, barriers such as stigma, lack of counseling services, long waitlists, and geographical barriers to access may hinder many college students from accessing psychological support. There is abundant evidence on the cost-effectiveness, scalability, and acceptability of e-mental health interventions, particularly supportive text and email interventions to address psychological distress and improve resilience and mental health literacy in youth and young adults. ResilienceNHope suite of text and email messaging programs has robust research evidence of effectiveness to reduce psychological treatment gaps at the population level, and may be effective in providing psychological support for college students globally. There is currently no other e-mental health program which specifically addresses youth mental health extensively and comprehensively and integrates mental health promotion, prevention, and intervention in one package, using youth-friendly delivery format. The components of ResilienceNHope have been evaluated to be effective and it is time to combine them into a more accessible package for further advanced evaluation among college students who experience significant transitions into early adulthood.

## Author contributions

BA and RS drafted the initial manuscript. All authors reviewed and revised the manuscript and approved the final version for submission.

## Funding

The ResilienceNHope research programs are funded by the Alberta Mental Health Foundation and the Royal Bank of Canada Foundation.

## Conflict of interest

VIOA is the founder and Principal Investigator of the ResilienceNHope messaging programs. VIOA is the Board Chair of the Global Psychological eHealth Foundation a not-for profit organization. BA is the President and Chief Executive Officer of the Global Psychological eHealth Foundation. VIOA and BA have no financial conflicts of interests in relation to this article. The remaining authors declares that the research was conducted in the absence of any commercial or financial relationships that could be construed as a potential conflict of interest.

## Publisher's note

All claims expressed in this article are solely those of the authors and do not necessarily represent those of their affiliated organizations, or those of the publisher, the editors and the reviewers. Any product that may be evaluated in this article, or claim that may be made by its manufacturer, is not guaranteed or endorsed by the publisher.
